# F231. GYM RATS: EXERCISE REVERSES COGNITIVE IMPAIRMENT IN THE PHENCYCLIDINE RAT MODEL OF SCHIZOPHRENIA

**DOI:** 10.1093/schbul/sby017.762

**Published:** 2018-04-01

**Authors:** Lisa Heaney, Joanna Neill, Antonio Gonzales, Alison Yung, Stuart Allan, Michael Harte

**Affiliations:** 1 University of Manchester; 2 University of Bath

## Abstract

**Background:**

The cognitive deficits of schizophrenia have been identified as an unmet clinical need. They are predictive of functional outcome [Green et al., 2000] and quality of life [Fujii et al., 2004], yet there are no treatments able to normalise cognition in schizophrenia. There is increasing evidence that exercise is helpful for these symptoms [Geyer et al., 2012], but the systems involved remain enigmatic. Animal models can be used to scrutinise both the behavioural and biological effects of exercise. The sub-chronic phencyclidine (PCP) rat model for schizophrenia is a well-established and widely utilised model that is used to investigate schizophrenia-like cognitive deficits [Neill et al., 201]. This two-part study investigates whether voluntary wheel running is able to reverse cognitive impairment in the sub-chronic PCP rat model for schizophrenia, and how long the effect of exercise lasts.

**Methods:**

Female Lister Hooded rats (n=80) were pseudo-randomised into four groups: vehicle-control; vehicle-exercise; PCP-control and PCP-exercise (n=20 per group). Rats were treated either with saline (vehicle) or PCP (2mg/kg, i.p. bi-daily, followed by a seven-day washout period). Vehicle and PCP exercise groups had access to a wheel for 1 hour a day, 5 days a week, for 6 weeks. The vehicle and PCP control groups were treated in the same way, but the wheels were locked. Rats were tested in the novel object recognition (NOR) memory paradigm pre-exercise (time point 1, T1) post-exercise (time point 2, T2), after two weeks rest (time point 3, T3) and four weeks rest (time point 4, T4). Half of the animals from each group (n=10 per group) were sacrificed post exercise (T2), and the remaining animals were sacrificed after 4 weeks rest. For each animal, 1 brain hemisphere was collected for protein analysis and 1 hemisphere was fixed for immunohistochemistry. Behavioural data were analysed using two-way ANOVA and post-hoc t-tests.

**Results:**

Pre-exercise (T1), in the retention phase both vehicle groups were spent more time exploring the novel over the familiar object, an effect that was not seen in the PCP groups. Post-exercise (T2 & T3), in the retention phase both vehicle groups and the PCP exercise group spent more time exploring the novel over the familiar object, an effect that was not seen in the PCP-control group. Post-exercise (T4) in the retention phase both vehicle groups were spent more time exploring the novel over the familiar object, an effect that was not seen in the PCP groups.

**Discussion:**

Exercise is able to rescue the NOR cognitive deficit seen in the sub-chronic rat model for schizophrenia. This corresponds with human studies reporting positive effects of exercise in patients with schizophrenia and provides a potential tool to thoroughly investigate the pro-cognitive effects of exercise The benefits of the exercise intervention were observed 2 weeks post-exercise with the deficits returning in the PCP treated animals when they were tested 4 weeks post-exercise. Post-mortem analysis is underway to determine the potential mechanisms by which exercise improves cognitive impairment.

